# Communication behavior of cognitively impaired older inpatients: A new setting for validating the CODEM instrument

**DOI:** 10.1007/s00391-019-01623-2

**Published:** 2019-10-18

**Authors:** Eva-Luisa Schnabel, Hans-Werner Wahl, Susanne Penger, Julia Haberstroh

**Affiliations:** 1 Network Aging Research, Heidelberg University, Bergheimer Straße 20, 69115 Heidelberg, Germany; 2 Interdisciplinary Ageing Research, Goethe University Frankfurt, Frankfurt, Germany

**Keywords:** Acute care hospital, Psychometrics, Psycholinguistics, Geriatric patients, Observational tool, Akutkrankenhaus, Psychometrie, Psycholinguistik, Geriatrische Patienten, Beobachtungsinstrument

## Abstract

**Background and objective:**

Acutely ill older patients with cognitive impairment represent a major subgroup in acute care hospitals. In this context, communication plays a crucial role for patients’ well-being, healthcare decisions, and medical outcomes. As validated measures are lacking, we tested the psychometric properties of an observational instrument to assess *Co*mmunication Behavior in *Dem*entia (CODEM) in the acute care hospital setting. As a novel feature, we were also able to incorporate linguistic and social-contextual measures.

**Material and methods:**

Data were drawn from a cross-sectional mixed methods study that focused on the occurrence of elderspeak during care interactions in two German acute care hospitals. A total of 43 acutely ill older patients with severe cognitive impairment (CI group, M_age_ ± SD = 83.6 ± 5.7 years) and 50 without cognitive impairment (CU group, M_age_ ± SD = 82.1 ± 6.3 years) were observed by trained research assistants during a standardized interview situation and rated afterwards by use of CODEM.

**Results:**

Factor analysis supported the expected two-factor solution for the CI group, i.e., a verbal content and a nonverbal relationship aspect. Findings of the current study indicated sound psychometric properties of the CODEM instrument including internal consistency, convergent, divergent, and criterion validity.

**Conclusion:**

CODEM represents a reliable and valid tool to examine the communication behavior of older patients with CI in the acute care hospital setting. Thus, CODEM might serve as an important instrument for researcher and healthcare professionals to describe and improve communication patterns in this environment.

## Zusammenfassung

**Hintergrund und Zielsetzung:**

Akut erkrankte Patienten mit kognitiver Beeinträchtigung stellen eine bedeutsame Subgruppe in Akutkrankenhäusern dar. In diesem Zusammenhang spielt Kommunikation eine entscheidende Rolle für das Wohlbefinden der Patienten, medizinische Entscheidungen und Outcomes. Da es an validierten Maßen mangelt, testeten wir die psychometrischen Eigenschaften eines Beobachtungsinstrumentes zur Erfassung des *Ko*mmunikationsverhaltens von *Dem*enzpatienten (KODEM) im Akutkrankenhaussetting. Dabei war es erstmalig möglich, linguistische und sozial-kontextuelle Maße einzubeziehen.

**Material und Methoden:**

Die Daten stammten aus einer querschnittlichen „Mixed-method“-Studie, deren Fokus auf dem Vorkommen von Elderspeak während Pflegeinteraktionen in zwei deutschen Akutkrankenhäusern lag. Insgesamt 43 akut erkrankte ältere Patienten mit schwerer kognitiver Beeinträchtigung (KB-Gruppe; M_Alter_ ± SD = 83,6 ± 5,7 Jahre) und 50 ohne kognitive Beeinträchtigung (KU-Gruppe; M_Alter_ ± SD = 82,1 ± 6,3 Jahre) wurden von geschulten Forschungsassistenten während einer standardisierten Interviewsituation beobachtet und anschließend mittels KODEM beurteilt.

**Ergebnisse:**

Die Faktorenanalyse bestätigte die erwartete Zwei-Faktoren-Lösung für die KB-Gruppe, d. h. einen verbalen Inhalts- und einen nonverbalen Beziehungsaspekt. Die Ergebnisse der vorliegenden Studie zeigten einwandfreie psychometrische Eigenschaften des KODEM-Instruments, was die interne Konsistenz, die konvergente, divergente und die Kriteriumsvalidität betrifft.

**Schlussfolgerung:**

KODEM erwies sich als ein reliables und valides Instrument zur Erfassung des Kommunikationsverhaltens von älteren Patienten mit KB im Akutkrankenhaussetting. Somit könnte KODEM als ein wichtiges Hilfsmittel für Forscher und Gesundheitspersonal dienen, um Kommunikationsmuster in dieser Umgebung zu beschreiben und zu verbessen.

## Introduction

Older adults with cognitive impairment are frequently admitted to acute care hospitals [28]. A representative study in Germany reported cognitive impairment in 40% of older inpatients [1]. There is strong evidence that hospitalization is associated with harmful consequences in this population, such as subsequent nursing home admission and mortality [28]. Therefore, becoming aware of unmet needs of cognitively impaired older patients, such as assistance in activities of daily living, hunger or thirst is a major public health issue in the acute care hospital setting due to substantial language impairments and challenging neuropsychiatric symptoms [6]. It is also well known that inefficient communication can negatively affect patients’ cooperation [39, 40], their well-being, [2] and healthcare decisions [13]; however, validated tools for measuring communication behavior in older patients with cognitive impairment in the acute care hospital setting are lacking so far.

### Communication with people with dementia: empirical and theoretical aspects

When explaining the communication behavior of people with dementia, three theoretical considerations deserve particular attention. First, communication has been defined as a context-dependent construct, which is closely related to well-being and distinct from functional linguistic skills of an individual [15, 23, 24]. In line with Watzlawick et al.’s widely acknowledged first axiom [35] “one cannot not communicate” (p. 30), it can be assumed that even patients with strongly impaired linguistic skills are able to communicate, albeit by other channels. Second, communication has been considered as a process that can be divided into four stages: presentation, attention, comprehension, and remembering [29]. This differentiation becomes important in people with dementia because distinct patterns of resources and deficits for each stage have been found [15]. At the level of presentation, for example, word finding failures, sentence fragments as well as reductions in grammatical complexity represent linguistic characteristics of people with dementia [22, 26]. Third, the second axiom of Watzlawick et al. [35] proposes a content versus a relationship aspect. The content aspect refers to the production and understanding of mainly verbal utterances; the relationship aspect refers to the underlying affective qualities of communication conveyed by mainly nonverbal stylistic (e.g., speech rate) and tonal features of communication (e.g., emotional tone of voice; [11]). The verbal content channel strongly declines in the course of dementia [22, 24, 26], whereas the nonverbal relationship channel can be preserved for a longer time [10, 24]. The ongoing functioning of the nonverbal relationship channel has also been supported by dementia-related challenging behavior occurring after the use of controlling tones [40]. Haberstroh et al. [15] integrated the aforementioned three theoretical considerations within the so-called TANDEM model to describe the communication behavior of people with dementia.

### Measurement issues and gaps

To date, there is a clear lack of suitable tools to measure communication behavior in people with dementia in a differentiated way. A shortcoming is that the existing tools operationalized communication rather as a functional skill and not as a context-dependent behavior related to well-being (for review, see [16]). Furthermore, previous instruments focused more on the verbal content than on the nonverbal relationship aspect. The CODEM instrument [24], an observational tool to assess Communication Behavior in Dementia, considers both the verbal content and the nonverbal relationship aspect inherent in communication behavior. In terms of previous CODEM validation efforts [23, 24], divergent validity was tested by correlations with the Barthel Index [27] examining functional performance in basic activities of daily living. Although functional status and communication behavior are considered as theoretically distinct constructs, there is an empirical overlap between both constructs in cognitively impaired older patients requiring other methods, such as factor analysis for determining divergent validity [3]. A further limitation of previous studies [23, 24] may be seen in the fact that linguistic features as well as social-contextual variables have not been considered as validation measures for CODEM so far. To date, the CODEM instrument has been validated in the nursing home and the ambulatory setting but not in the acute care hospital setting. Given that older patients with cognitive impairment meanwhile play a relatively prominent role in acute care, this is an important missing link in the existing CODEM literature.

### Objectives and hypotheses

The current study aims to address this gap and to examine the psychometric properties of CODEM as a diagnostic and interventional tool in the acute care hospital setting, particularly for use in patients with severe cognitive impairment. As a novel feature, verbal and nonverbal linguistic features as well as social-contextual variables will be included in the validation analysis.

In line with previous research conducted in the nursing home setting [24], we assume to find support for a two-factor solution representing a verbal content and a nonverbal relationship component of communication, with higher ratings for the relationship compared to the content aspect in severely cognitively impaired (CI) patients but not in cognitively unimpaired (CU) patients. In terms of linguistic indicators, we expect moderate to strong correlations with patients’ linguistic features in terms of sentence length and speech rate. Regarding divergent validity, we expect low correlations with subjective hearing capacity that captures the sensory loss of an individual and not necessarily the communication behavior when compensatory strategies are used [14]. We also expect low correlations between verbal memory recall and the nonverbal relationship aspect. With respect to social-contextual variables, we assume that nurses’ emotional tone is more strongly associated with the nonverbal relationship aspect when compared to the verbal content aspect as conveying affective information.

## Methods

### Recruitment

The data were part of a larger cross-sectional study on elderspeak in the acute care hospital setting conducted from September 2017 to March 2018. Detailed information on the study design and recruitment procedure can be found elsewhere [32]. Briefly, participants were recruited from a general internal medicine ward (*n* = 36 beds, mean length of stay = 4.9 days) and a geriatric ward (*n* = 35 beds; mean length of stay = 16.5 days) of two acute care hospital settings (*n* = 114 and 105 beds, respectively). Both hospitals were affiliated with the university located in the city center of a medium-sized town in southwest Germany. A two-month internship by the first author in both hospitals served to prepare the assessments. The study was approved by the local ethics committee of the Faculty of Behavioral and Cultural Studies at Heidelberg University in July 2017, as well as by hospital staff leadership and staff councils.

All registered nurses were eligible for study inclusion. Inclusion criteria for patients were a minimum age of 65 years and CI in 50% of the patient sample. Allocation to the CI group was based on the 10/11 cut-off of the 6‑Item Cognitive Impairment Test (6CIT [17]) covering the domains orientation, calculation, and verbal memory recall. This tool was chosen because it represents a validated and time-efficient screening instrument in the acute care hospital setting with higher sensitivity (sensitivity and specificity 0.88 and 0.95, respectively) compared to medical records [17]. As a dementia diagnosis was only partially available, the more cautious term CI instead of dementia will be used in the following. Exclusion criteria were terminal illness, isolation, insufficient German language skills, and impending discharge. Patients of the wards were screened for the abovementioned eligibility criteria using the patient lists, medical records, and consulting nurses. All participants or legal representatives of CI patients included in the study as well as all individuals in the audio recording room (e.g., co-patients, nursing aides) had to provide written informed consent (WIC) prior to the assessments. Approximately 27% of the screened patients participated in the study resulting in a sample of 106 patients (49% with CI, 6CIT error scores: mean = 10.8, *SD* = 8.6, range = 0–28). In total, 34 registered nurses who were responsible for the respective patient rooms took part in the study. This corresponds to the precalculated sample size of at least 50 patients (50% with CI) per hospital setting. For further information on sampling see [32].

### Observational procedure and sample

In this study, three different data sources were used: (a) audio recordings during the morning or evening care, (b) standardized interviews with patients and nurses, and (c) extracting basic patient information from the medical information system. The linear Pulse Code Modulation (PCM) digital audio recorders (48 kHz, 16 bits) located in the patient rooms were immediately started before the nurse entered the room. Each patient was only recorded once, whereas 76% of the nurses were recorded several times but not more than six times.

Patients’ communication behavior was examined by three trained psychology students and one sociology student via the CODEM instrument [24]. The training was performed in the field based on the manual of Kuemmel et al. [24]. Research assistants conducted standardized interviews with patients while they observed their communication behavior. Interviews focused on sociodemographic, health and hospital-related variables as well as cognitive status. Immediately after the standardized observational situation (*M* = 21 min), interviewers rated patients’ communication behavior within three minutes; however, interviews were not feasible in 10% of the patients due to refusal, transfers, or advanced stages of CI and thus the evaluation of CODEM was also not possible. Furthermore, CODEM data were missing for the first two pilot trials. In total, observational data on patients’ communication behavior were available for a sample of 43 CI patients and 50 CU patients associated with a sample of 31 nurses. As can be seen in Table 1, CI patients did not differ from CU patients in basic sociodemographic, health, hospital-related and contextual variables; however, CI patients showed prototypical differences in terms of significantly lower communication behavior, lower cognitive and functional status as well as reduced linguistic skills. Nurses’ characteristics of the analyzed sample are displayed in Table 2.

**Table 1:** Patient characteristics (*N* = 93)

	CI (*n* = 43)			CU (*n* = 50)			*p*-value
	*M*	*SD*	%	*M*	*SD*	%	
Age (years)	83.6	5.7	–	82.1	6.3	–	0.212
Gender (female/male)	–	–	51/49	–	–	56/44	0.641
Mother tongue (German/non-German)	–	–	95/5	–	–	96/4	0.858
Lower/intermediate/upper secondary school	–	–	62/23/15	–	–	71/10/19	0.297
Private/nursing/retirement/residential home	–	–	87/10/0/3	–	–	92/0/2/6	0.090
Hospital (general/geriatric)	–	–	53/47	–	–	54/46	0.961
Shift (morning/evening)	–	–	56/44	–	–	38/62	0.086
Length of hospital stay (days)	14.9	7.4	–	13.3	6.6	–	0.271
Admission to examination (days)	7.3	6.5	–	6.4	4.9	–	0.432
CODEM (total mean score; 0–5)^a^	3.2	1.1	–	4.8	0.2	–	**<0.001**
Cognitive status (6CIT error sum scores; 0–28)^b^	19.0	5.3	–	3.9	3.1	–	**<0.001**
Functional status (sum scores; 0–100)^c^	48.6	26.0	–	75.9	23.3	–	**<0.001**
Subjective hearing capacity (1–5)^d^	2.8	1.0	–	2.7	1.0	–	0.696
Speech rate (words per min)	122.3	32.8	–	146.5	23.4	–	**<0.001**
Mean length of utterances (words per utterance)^e^	2.4	0.7	–	3.1	0.9	–	**<0.001**

*p* values for interval-scaled variables from *t*-tests and for dichotomous variables from χ^2^-tests; significant *p* values are in boldface

*CI *severely cognitively impaired patients (6CIT >10), *CU *cognitively unimpaired patients (6CIT ≤10), *M* mean, *SD* standard deviation

^a^*CODEM* observational tool to assess the frequency of communication behavior in dementia [24] ranging from 0 (never) to 5 (always)

^b^*6CIT* 6‑Item Cognitive Impairment Test [17]; lower error scores indicate a better cognitive status

^c^*Barthel Index * [27]; higher values indicate a better functional status

^d^Single item [34] ranging from 1 (very good) to 5 (very poor)

^e^Segmentation into utterances (i.e., syntactic units) was based on German guidelines [36]

**Table 2:** Nurse characteristics (*N* = 31)

	*M*	*SD*	%
Age (years)	39.2	12.5	–
Gender (female/male)	–	–	84/16
Mother tongue (German/non-German)	–	–	63/37
Lower/intermediate/qualification for applied upper secondary studies/upper secondary school	–	–	3/47/27/23
Registered nurse/geriatric trained nurse	–	–	73/27
Experience as a nurse (<5/5–10/11–15/>15 years)	–	–	23/30/3/44

### Measures

#### Communication behavior, functional and sensory indicators.

The CODEM instrument consists of 15 items rated on a 6-point Likert scale (0 = never and 5 = always). Higher values indicate a higher frequency of communication behavior. Previous exploratory and confirmatory factor analyses [24] revealed two subscales: verbal content and nonverbal relationship aspects. Previous reliability analysis showed an excellent internal consistency (Cronbach’s alpha = 0.95), whereas construct validity in terms of convergent and divergent validity revealed high correlations for both constructs (*r* = 0.88 for communication abilities and 0.63 for functional status).

Patients’ functional status in this and previous studies was evaluated by nurses using the Barthel Index [27]. Patients’ subjective hearing capacity was operationalized by a well-established [34] single item (“how would you rate your current hearing capacity?”) ranging from 1 (very good) to 5 (very poor). Visual acuity was not assessed because it is more related to the use of the physical environment than to social communication [34].

#### Linguistic and social-contextual indicators.

Well-established verbal and nonverbal linguistic measures [20, 21, 26] were extracted for patients as well as for nurses. As a nonverbal stylistic feature of the voice, the speech rate was quantified as words per min rate using the FOLKER transcription tool [31]. As a verbal feature, syntactic complexity was operationalized by the mean length of utterances [21]. Segmentation into utterances (i.e., syntactic units) was based on German guidelines for spoken language interactions [36]. In accordance with magnitudes used in previous studies [37, 39], 10% of the data (*n* = 926 utterances for patients, *n* = 1455 utterances for nurses) were independently processed by two trained individuals. Segmentation agreement was determined by the chance-corrected Thomann method using the segmentation agreement calculator in ELAN [25]. The degree of agreement was high for patients’ (88%) and nurses’ (86%) utterances. To assess the underlying affective qualities of social communication in terms of a controlling and a person-centered tone of voice, nurses’ emotional tone was judged by naïve raters using the two subscales (Cronbach’s alpha = 0.98 for both) of the Emotional Tone Rating Scale [38]. Detailed information on the rating procedure can be found elsewhere [32].

### Data analysis

Psychometric testing was only performed in the target group of CI patients as the CU group exhibited strong ceiling effects (i.e., highest possible CODEM score) varying between 62% and 96% across all items. Data analyses were conducted by IBM SPSS version 25 (Armonk, NY, USA). Missing values for single items of the CODEM occurred only in 2% of the participants resulting in a total sample of 42 CI patients for the factor and the reliability analyses.

#### Exploratory factor analysis.

In order to test the underlying factor structure of the CODEM as applied in this completely new setting, an exploratory factor analysis (EFA) was conducted using a principal component analysis with oblique (Promax) rotation (*κ* = 4) due to expected correlations between factors [24]. The Kaiser-Meyer-Olkin (KMO) procedure [19] supported that data for the CI group were appropriate for conducting a factor analysis (KMO ≥0.8). The number of factors was tested by the Kaiser’s eigenvalue >1 criterion [18] and the scree test [4].

#### Reliability.

As an indicator of reliability, internal consistency was measured by Cronbach’s alpha for both subscales separately. Interpretation was based on established rules of thumb: alpha > 0.9 for excellent, alpha > 0.8 for good, alpha > 0.7 for acceptable, alpha > 0.6 for questionable, and alpha > 0.5 for poor reliability [12]. Furthermore, corrected item-total correlations (ITCs) were examined to identify items that did not sufficiently contribute to the respective subscale.

#### Validity.

Given the results of the factor and reliability analyses, mean scores were calculated for both subscales as well as a total mean score. For construct validity testing, Spearman correlations with convergent (patients’ linguistic indicators) and divergent (subjective hearing capacity, verbal memory recall) measures as well as with social-contextual variables (nurses’ linguistic indicators, time of day) were computed. Spearman correlations were chosen because normal distribution was not given for all variables. The effect sizes of correlation coefficients were interpreted as follows: 0.10 small, 0.30 medium and 0.50 large [7]. Differences in the magnitude of the associations with the content versus the relationship aspect of communication were examined by testing the difference between two dependent correlations based on Fisher’s *r*-to-*z* transformations [9]. To control for multiple pairwise comparisons, the Bonferroni-Holm correction was used.

For criterion validity testing, differences in communication behavior between CI and CU patients were examined using an analysis of variance with repeated measurements, with the aspect of communication (content versus relationship) as a within-subject factor and cognitive group (CI versus CU) and hospital setting (general versus geriatric) as between-subject factors. As effect size indicator, partial eta squared (η_p_^2^) was used (0.01 small effect, 0.06 medium effect, 0.14 large effect [8]).

## Results

### Factorial structure

Both the Kaiser’s criterion and the scree test supported a two-factor solution in the CI sample as found by [24]. As expected, both factors were strongly correlated (*r* = 0.69). The total explained variance was 74%. The Promax rotated matrix of factor loadings and the communalities also confirmed the expected patterns (Table 3). In line with previous research [24], the first factor was labelled “content aspect” and the second factor “relationship aspect”.

**Table 3:** Results of the exploratory factor analysis and reliability statistics for severely cognitively impaired patients (*n* = 42)

Items	Rotated factor loadings			Item reliability
	Factor 1 Content	Factor 2 Relationship	Communality	ITCs
*Presentation*				
03. She/he uses a sensible sentence structure	**0.70**	0.26	0.81	0.86
04. She/he uses words according to their meaning	**0.66**	0.23	0.70	0.80
05. She/he comes up with the right words	**0.64**	0.23	0.67	0.77
01. She/he signalizes the need to communicate	0.27	**0.61**	0.67	0.76
02. She/he shows interest in the interaction partner	0.02	**0.89**	0.81	0.86
06. She/he shows emotions	−0.16	**1.01**	0.82	0.83
*Attention*				
07. She/he can make eye contact	−0.22	**0.93**	0.63	0.67
08. She/he maintains eye contact appropriately	0.23	**0.61**	0.61	0.72
*Comprehension*				
09. She/he understands complex questions and sentences	**1.01**	−0.18	0.81	0.83
10. She/he responds sensibly to what is said	**1.04**	−0.23	0.81	0.82
11. She/he demonstrates appropriate nonverbal responses to what is said	0.33	**0.60**	0.74	0.79
12. She/he reacts to the feelings of the other	0.11	**0.83**	0.82	0.87
*Remembering*				
13. She/he performs the task independently	**0.81**	0.13	0.81	0.86
14. She/he communicates without memory aids from the other	**0.80**	0.10	0.77	0.83
15. She/he remains on an issue	**0.82**	−0.04	0.63	0.73
*Factor statistics*				
Cronbach’s alpha (CI 95%)	0.95 (0.92, 0.97)	0.93 (0.90, 0.96)	–	–
Initial eigenvalue	9.65	1.45		
Initial variance (%)	64.36	9.66		

Factor analysis using principal component analysis with oblique (Promax) rotation revealed a two-factor solution (content and relationship aspect of communication), explaining 74% of the variance. *Bold letters* indicate the highest standardized factor loadings for each item. One patient was excluded from factor analysis due to a missing CODEM item resulting in a sample of 42 severely cognitively impaired patients

*ITCs* corrected item-total correlations, *CI *95% confidence interval

### Reliability

Internal consistency reliability and ITCs for CI patients are displayed in Table 3. Subscale reliability coefficients indicated excellent reliability for both subscales (alpha = 0.95 for the content aspect and alpha = 0.93 for the relationship aspect). The ITCs ranged between* r* = 0.73 and 0.86 for the content aspect and between* r* = 0.67 and 0.87 for the relationship aspect indicating high discriminatory power of the items for both subscales. With respect to the different hospital settings, internal consistency for both subscales did not differ between the general and the geriatric acute care hospital.

### Validity

The examination of convergent validity showed moderate to strong correlations between patients’ linguistic indicators and CODEM scores of comparable magnitude for the verbal content and the nonverbal relationship aspect (Table 4). In terms of divergent indicators, correlations with subjective hearing capacity and verbal memory recall were relatively low, particularly between verbal memory recall and the relationship aspect (*r* = −0.08). With respect to social-contextual variables, the evening shift was associated with an increased nonverbal communication behavior of patients; however, nurses’ verbal and nonverbal linguistic indicators were not substantially associated with CODEM dimensions with the exception of a moderately high correlation between the mean length of utterances and the verbal content component. Nurses’ emotional tone was also not significantly correlated with the relationship aspect. The correlations with patients’ functional status were also checked and were substantial for both CODEM components (*r* > 0.5, *p* < 0.001).

**Table 4:** Construct validity: Spearman correlations of CODEM (total and subscales) with convergent, divergent, and social-contextual constructs for severely cognitively impaired patients (*n* = 43)

Measures	*n*	*M*		*SD*	Level	CODEM _total_ (*r*)	CODEM _content_ (*r*)	CODEM _relationship_ (*r*)	Corrected *p-*value
*Convergent validity*									
Speech rate (words per min)	43	122.3		32.8	Patients	0.51**	0.50**	0.49**	1
Mean length of utterances (in words)	43	2.4		0.7	Patients	0.38*	0.36*	0.40**	1
*Divergent validity*									
Subjective hearing capacity (1–5)^a^	36	2.8		1.0	Patients	−0.14	−0.12	−0.16	1
Verbal memory recall (6CIT_error scores;_ 0–10)^b^	43	8.2		2.4	Patients	−0.17	−0.21	−0.08	0.868
*Social-contextual constructs*									
Speech rate (words per min)	43	156.1		22.3	Nurses	0.18	0.21	0.15	1
Mean length of utterances (in words)	43	3.3		0.7	Nurses	0.24	0.33*	0.12	0.256
Controlling tone of voice (1–5)^c^	38	2.5		0.6	Nurses	−0.19	−0.14	−0.25	1
Person-centered tone of voice (1–5)^d^	38	3.6		0.5	Nurses	0.02	−0.01	0.09	1
Shift (morning/evening)	24/19	–		–	Organization	0.12	−0.01	0.31*	**0.027**

Variables describing features on the patient, nurses, and organizational level are displayed;* p‑*values refer to differences in the magnitude of associations for the verbal versus the relationship aspect adjusted by the Bonferroni-Holm correction for multiple univariate comparisons; significant *p-*values after correction are in *boldface*

^a^Single item [34] ranging from 1 (very good) to 5 (very poor)

^b^*6CIT* 6‑Item Cognitive Impairment Test [17]; lower error scores indicate a better verbal memory recall

^c,d^Mean emotional tone ratings of naïve judges [32] ranging from 1 (not at all) to 5 (very)

*n* varies due to ^(a)^ difficulties to answer the question or ^(c,d)^ not fulfilling criteria for rating procedure

*p < 0.05, **p < 0.01, ***p < 0.001

**Fig. 1:**
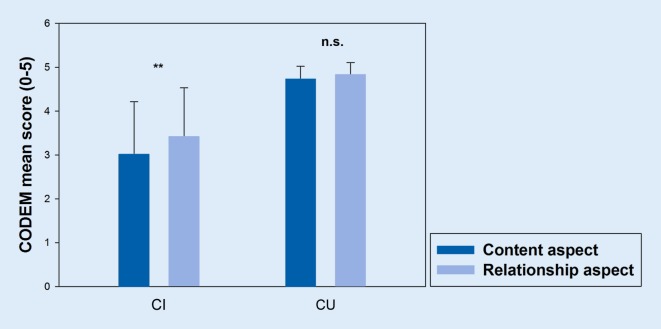
Mean differences in the frequency of communication behavior between severely cognitively impaired (*CI*, *n* = 43) and cognitively unimpaired (*CU*; *n* = 50) patients for the content and the relationship aspect ranging from 0 (never) to 5 (always). Higher values indicate a higher frequency of communication behavior. Standard deviations are represented by error bars. *CODEM* observational tool to assess communication behavior in dementia (*n.s.* not significant, **p < 0.01)

As can be seen in Fig. 1, mean ratings for CU patients were consistently higher when compared to CI patients. In fact, the ANOVA revealed a significant main effect of cognitive group on communication behavior (*F*[1,89] = 97.16, *p* < 0.001, η_p_^2^ = 0.522). The main effect of hospital setting on communication behavior was not significant (*p* = 0.589), indicating similar patterns for both hospital settings. Importantly, criterion validity was confirmed by a significant interaction effect between cognitive group and communication aspect (*F*[1,89] = 5.46, *p* = 022, η_p_^2^ = 0.058). Post hoc dependent t‑tests using bootstrapping procedures to estimate the bias-corrected and accelerated 95% confidence interval (BCa 95% CI) showed that the difference between the content and relationship aspect (−0.40, BCa 95% CI [−0.64, −0.16]) was significant for CI patients (*t*[42] = −3.30, *p* = 0.002) but not for CU patients (*t*[49] = −1.90, *p* = 0.062). As evident from Fig. 1, ratings for the relationship aspect were higher than for the content aspect in CI patients.

## Discussion

To our knowledge, this is the first study that tested the psychometric properties of the CODEM instrument for use in the acute care hospital setting. Considering linguistic as well as contextual variables was also a novel step compared to previous CODEM validation studies [23, 24]. The current study was able to show that communication behavior can also be assessed in a psychometrically sound way in acutely ill older patients with CI.

Exploratory factor analysis supported the fit of the previously found two-factor solution [24] for the acute care hospital setting reflecting a verbal content and a nonverbal relationship aspect of communication. Comparing both factors also revealed typical patterns [23, 24] with higher ratings for the relationship aspect when compared to the content aspect in the CI group but similarly high ratings for both aspects in the CU group. The strong ceiling effects in the CU group suggest that CODEM may be a useful and informative measure in CI patients but does not provide additional benefit in CU patients.

With respect to validity testing, the different indicators provided support for convergent and divergent validity of CODEM. As expected, associations with patients’ linguistic indicators showed moderate to strong effect sizes, whereas associations with divergent measures were relatively low. In line with previous research [23, 33], correlations between verbal memory recall and the relationship aspect were relatively low when compared to other variables, which are more strongly related to language than to memory. With respect to differences in the strength of associations with the verbal content versus the nonverbal relationship aspect, verbal and nonverbal linguistic features in terms of mean length of utterances and speech rate were not differentially associated with both aspects. An explanation for this finding might be that both measures contain verbal as well as nonverbal elements. For example, speech rate is considered as a nonverbal stylistic measure [11] but likewise depends on the number of words. Surprisingly, support for the assumption that nurses’ emotional tone is more strongly associated with the relationship aspect could not be found. Most of the nurses’ measures were not significantly correlated with the CODEM factors. An explanation may rely on previous findings in the acute care hospital setting indicating that factors such as the salient functional status of patients might play a more important role in eliciting nurses’ communication behavior than cognitive impairment per se [32]. With respect to social-contextual variables, patients’ nonverbal communication behavior increased during the evening shift. This finding might find at least a partial explanation by the sundown syndrome coming along with challenging behavior [5].

From a practical point of view, the findings suggest that CODEM could be a promising measure to describe and improve communication patterns in the acute care hospital setting. With respect to diagnostic issues, CODEM allows communication resources and deficits of acutely ill older patients to be detected at different stages of the communication process. This may enable hospital staff to accommodate their communication behavior in a specific manner leading to more efficient and enriching social interactions. Shifting the focus from verbal to nonverbal communication behavior may also raise the awareness of essential current needs of older CI patients. The identification of unmet needs is highly important in this vulnerable sample due to the linkage with negative cognitive-affective states and neuropsychiatric symptoms [6]. In line with previous research [23, 24], CODEM was shown to be a largely feasible and time-efficient instrument to examine the communication behavior of CI patients. In past research, the training was not only successful for observing research assistants [23] but also for observing nurses [24]. Thus, CODEM might be easily implemented into the hospital routines by combining the observational phase with established screening procedures. The rating process per se requires only three minutes.

With respect to interventional issues, the relationship aspect as a crucial resource of CI patients might serve not only as an important patient outcome for future psychosocial interventions but also as an indicator of the quality of hospital care due to its linkage with well-being. In fact, first psychosocial intervention studies supported that individual music therapy is able to stimulate the nonverbal relationship channel by increasing the communication behavior, well-being, and positive affects of people with advanced dementia [30]. Future research should explore whether reductions of elderspeak features, such as controlling tones of nurses’ voice can facilitate positive nonverbal communication behavior of CI patients in acute care hospitals.

### Limitations

Limitations of the current study are the relatively small sample size based on two acute care wards and the lack of a standardized interview situation in some patients. Nevertheless, this study was able to replicate earlier findings and to link the observational findings with innovative linguistic and social-contextual data. Another limitation is that divergent measures were based on single items; however, previous studies indicated that subjective hearing capacity can reliably be assessed by a single item, even in multimorbid older adults [34]. Furthermore, the present study did not assess interrater and retest reliability for CODEM as the primary focus was on elderspeak; however, raters underwent standardized training based on earlier manuals and received supervision at the beginning. Interrater and retest reliability were shown to be high in previous research [23, 24]. Finally, this study was only able to differentiate between a verbal content versus a nonverbal relationship aspect because the shorter version of the CODEM instrument developed for use in nursing home settings was applied [24]. Although the relationship aspect refers to the nonverbal channel of communication, it does not capture nonverbal content aspects such as reactions to gestures or pictures [23].

## Practical conclusion

- The CODEM instrument is a largely feasible and easily applicable instrument to assess the verbal and nonverbal communication behavior of older patients with CI in the acute care hospital setting.
- CODEM enables the examination of communication in terms of behavior that is relevant for well-being.
- Applying CODEM does not require more than three minutes when combined with established screening routines.
- CODEM revealed sound psychometric properties including internal consistency, convergent, divergent, and criterion validity.
- CODEM might serve as an important diagnostic and interventional tool for acutely ill older patients with CI if it is administered by trained hospital staff.
- Further studies including larger samples and a more heterogeneous set of acute care hospital settings are required.
